# Cobalt/copper-decorated carbon nanofibers as novel non-precious electrocatalyst for methanol electrooxidation

**DOI:** 10.1186/1556-276X-9-2

**Published:** 2014-01-03

**Authors:** Nasser A M Barakat, Mohamed El-Newehy, Salem S Al-Deyab, Hak Yong Kim

**Affiliations:** 1 Department of Organic Materials and Fiber Engineering, College of Engineering, Chonbuk National University, Jeonju 561-756, South Korea; 2 Chemical Engineering Department, Faculty of Engineering, Minia University, El Minia 61111, Egypt; 3 Petrochemical Research Chair, Department of Chemistry, College of Science, King Saud University, Riyadh 11451, Saudi Arabia; 4 Department of Chemistry, Faculty of Science, Tanta University, Tanta 31527, Egypt

**Keywords:** Non-precious catalyst, Electrospinning, Fuel cells, Decorated carbon nanofibers, Methanol electrooxidation

## Abstract

In this study, Co/Cu-decorated carbon nanofibers are introduced as novel electrocatalyst for methanol oxidation. The introduced nanofibers have been prepared based on graphitization of poly(vinyl alcohol) which has high carbon content compared to many polymer precursors for carbon nanofiber synthesis. Typically, calcination in argon atmosphere of electrospun nanofibers composed of cobalt acetate tetrahydrate, copper acetate monohydrate, and poly(vinyl alcohol) leads to form carbon nanofibers decorated by CoCu nanoparticles. The graphitization of the poly(vinyl alcohol) has been enhanced due to presence of cobalt which acts as effective catalyst. The physicochemical characterization affirmed that the metallic nanoparticles are sheathed by thin crystalline graphite layer. Investigation of the electrocatalytic activity of the introduced nanofibers toward methanol oxidation indicates good performance, as the corresponding onset potential was small compared to many reported materials; 310 mV (vs. Ag/AgCl electrode) and a current density of 12 mA/cm^2^ was obtained. Moreover, due to the graphite shield, good stability was observed. Overall, the introduced study opens new avenue for cheap and stable transition metals-based nanostructures as non-precious catalysts for fuel cell applications.

## Background

The extensive use of fossil fuels is causing environment pollution and global warming problems. Fuel cell is a good technological option for solving energy and pollution problems. Polymer electrolyte membrane fuel cells (PEMFCs) have been investigated as high-density power sources in automobiles and in microelectronics. The efficiency of fuel cells depends on the catalytic activity of the catalysts. The use of methanol as a fuel is getting popular because it is a liquid which can be easily stored and handled. Methanol is also easier to supply to the public using our current infrastructure. In the DMFCs, methanol is directly oxidized to carbon dioxide and water, providing a new way to store and convey the energy [1-3]. The successful commercialization is quite dependent on the cost, activity, and durability of the electrocatalysts [3,4]. At present, almost all pre-commercial low-temperature fuel cells use Pt-based electrocatalysts [5-8]. Accordingly, the manufacturing cost is relatively high which constrains their wide applications. Moreover, the catalyst poisoning by CO or CHO species is another real problem facing most of the Pt-based electrocatalysts [3,9,10]. Compared to the precious metals, transition metals are abundant and very cheap. Among the transition metals, cobalt has a well-known catalytic activity in many chemical reactions. It is also used as a cocatalyst to annihilate the Pt poisoning [11]. Beside cobalt, copper-based materials also show good performance as electrocatalysts [12,13].

In the DMFCs, methanol is oxidized to carbon dioxide at the anode according to the reaction: CH_3_OH + H_2_O = CO_2_ + 6H^+^ + 6e. The reaction is considered to be a combination of adsorption and electrochemical reaction on the anode surface [14,15]. Accordingly, because of the adsorption capacity of carbon, it was exploited to enhance the electrocatalytic activities for many electrodes [16-20]. Addition of alloying elements to Cu has been demonstrated to provide increased electrocatalytic activity in comparison to the pure Cu electrode. For example, MnCu alloy shows a much improved electrochemical activity for the oxidation of glucose in alkaline media in comparison to that of the pure Cu electrode [21].

Nanofibers have gained much prominence in the recent years due to the heightened awareness of their potential applications in many fields including textiles, chemical synthesis, medicine, engineering, and defense. Among several methods for nanofiber production, electrospinning is the most widely used technique due to simplicity, high yield, effectiveness and low-cost aspects [22-24]. It is noteworthy mentioning that as compared to nanoparticles, the large axial ratio provides the nanofibrous catalysts a distinct advantage especially when utilizing in the electron transfer-based processes [25,26]. Carbon nanofibers (CNFs) prepared by the electrospinning process gradually attracted the attention of most researchers because of the associated advantages of the synthesizing technique and the obtained product. Moreover, it is easy to control the morphology and pore structure of the obtained nanofibers [27]. Currently, CNFs are widely used in many fields such as hydrogen energy [28,29], electrochemical capacitors (EDLCs) [30], lithium-ion rechargeable batteries (LIBs) [31], and fuel cells [32].

Poly(vinyl alcohol) (PVA) is a semi-crystalline compound with comparatively high carbon content (*ca*. 54.5%), and easily splits into hydroxyl groups in the polymer chain which makes it favorable for use as a precursor for the production of the carbonaceous materials; however, low yield is the main constrain. The low decomposition temperature of PVA is the main reason for the low carbonization yield [33]. Therefore, the researchers are focusing on enhancing the thermal stability of PVA. Some strategies were introduced such as dehydration of PVA from 100°C to 290°C under tension in a mixed gas atmosphere [34], preoxidation or subsequent dehydration [35], and using iodine as a stabilizer for PVA to promote dehydrogenative polymerization during the carbonization process [36]. Recently, it was reported that cobalt strongly enhances the graphitization of PVA [22,37].

Therefore, the main goal of this study is to introduce a new non-precious catalyst with good electrocatalytic activity. Enhancement of the activity will be based on exploiting the influence of synergetic effect of cobalt and copper bimetallic nanoparticles as well as the advantage of the nanofibrous morphology. In this study, Co/Cu-decorated carbon nanofibers are introduced as novel non-precious catalyst for methanol electrooxidation. The introduced nanofibers have been synthesized by calcination of electrospun nanofibers composed of cobalt acetate, copper acetate, and PVA in argon atmosphere at 750°C. The obtained nanofibers revealed good performance as electrocatalyst for methanol oxidation.

### Experimental section

Solution containing copper acetate monohydrate (CuAc, 99.9 Sigma-Aldrich Corporation, St. Louis, MO, USA), cobalt acetate tetrahydrate (CoAc, 98% assay Junsei Chemical Co., Ltd, Japan), and poly(vinyl alcohol) (PVA) (10 wt.% concentration in water, molecular weight (MW) = 65,000 g/mol; DC Chemical Co, Ltd, Seoul, South Korea) was electrospun at a voltage of 20 kV using a high-voltage DC power supply. Typically, CuAc (25 wt.%) and CoAc (25 wt.% ) aqueous solutions were mixed with PVA solution to form a mixture containing 80 wt.% polymer and Cu/Co mass ratio of 1:4. The final solution was stirred at 50°C for 5 h and then subjected to the electrospinning process at 20 kV. The formed nanofiber mats were initially dried for 24 h at 80°C under vacuum and then calcined at 750°C for 5 h in argon atmosphere with a heating rate of 2.3°C/min.

The surface morphology was studied by scanning electron microscope (SEM) (JEOL JSM-5900; JEOL Ltd, Tokyo, Japan) and field-emission scanning electron microscope equipped with energy-dispersive X-ray (EDX) analysis tool (field emission scanning electron microscopy, FESEM; Hitachi S-7400, Japan). Information about the phase and crystallinity was obtained by using Rigaku x-ray diffractometer (XRD; Rigaku Corporation, Tokyo, Japan) with Cu Kα (λ = 1.540 Å) radiation over Bragg angle ranging from 10° to 90°. High-resolution image and selected area electron diffraction patterns were obtained with transmission electron microscope (TEM) (JEOL JEM-2010, JEOL Ltd, Tokyo, Japan) operated at 200 kV equipped with EDX analysis. The Raman spectra were measured using Nanofinder 30 spectrometer (Tokyo Inst. Co., Machida-shi, Tokyo, Japan) equipped with a He/Ne (λ = 633 nm laser) and the scattering peaks were calibrated with a reference peak from a Si wafer (520 cm^-1^). Raman spectra were recorded under a microscope with a × 40 objective in range of 0–1,600 cm^-1^ and 3 mW of power at the sample. The electrochemical measurements were performed in a 1 M KOH solution at room temperature. The measurements were performed on a VersaSTAT 4 (USA) electrochemical analyzer and a conventional three-electrode electrochemical cell. A Pt wire and an Ag/AgCl electrode were used as the auxiliary and reference electrodes, respectively. Glassy carbon electrode was used as working electrode. Preparation of the working electrode was carried out by mixing 2 mg of the functional material, 20 μL of Nafion solution (5 wt.%) and 400 μL of isopropanol. The slurry was sonicated for 30 min at room temperature. An amount of 15 μL from the prepared slurry was poured on the active area of the glassy carbon electrode which was then subjected to drying process at 80°C for 20 min. All potentials were quoted with regard to the Ag/AgCl electrode. High-purity nitrogen gas was used before and during measurements to deaerate the electrolyte in all measurements. Normalization of the current density was achieved based on the surface area of the utilized glassy carbon electrode (0.07 cm^2^).

## Results and discussion

**Figure 1:**
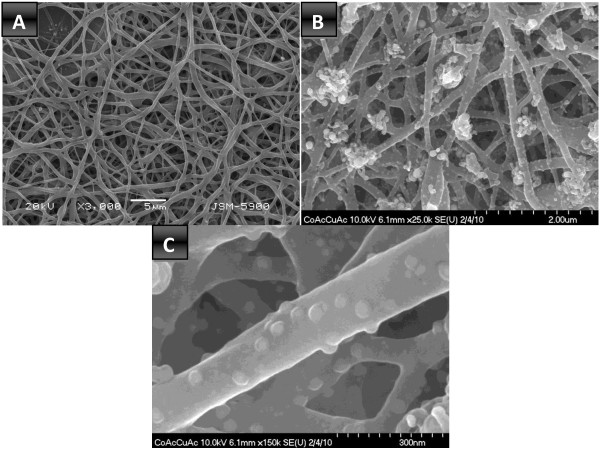
**SEM and FE SEM images for the electrospun and sintered nanofibers. (A)** SEM images for the electrospun nanofibers. **(B,C)** SEM and FE SEM images of the sintered nanofibers, respectively.

Electrospinning technique involves the use of a high voltage to charge the surface of a polymer solution droplet and thus to induce the ejection of a liquid jet through a spinneret. Due to bending instability, the jet is subsequently stretched by many times to form continuous ultra-thin fibers. Accordingly, many functional nanofibers have been synthesized by using the electrospinning process. It is noteworthy mentioning that inorganic nanofibers can be synthesized by electrospinning of a sol–gel composed of a polymer and a proper metallic precursor. However, the selected metallic precursor should have polycondensation tendency. Polycondensation of the utilized precursor is the main characteristic to get the sol–gel formulation. Typical metallic precursors are metal alkoxides, which undergo hydrolysis and polycondensation reactions to form an integrated network [38]. Some metal salts such as chloride, nitrate, and acetates can be hydrolyzed and polycondensate to form the gel networks. In this study, copper acetate (CuAc) and cobalt acetate (CoAc) were utilized as metallic precursors due to the good condensation character as shown in this equation [39]:

where M is Co or Cu. PVA was chosen for two functions: optimization of the sol–gel viscosity and as a source of carbon due to the positive impact of CoAc on PVA graphitization [22,37]. Actually, the obtained CoAc/CuAc/PVA electrospun nanofibers have good nanofibrous morphology as shown in Figure 1A. The calcination of the obtained electrospun nanofibers in argon atmosphere did not strongly affect the nanofibrous morphology as shown in Figure 1B. However, there is a distinct change in the nanofibers' surface after calcination; some nanoparticles are observed attaching to the surface of the sintered nanofibers as shown in Figure 1C.

**Figure 2:**
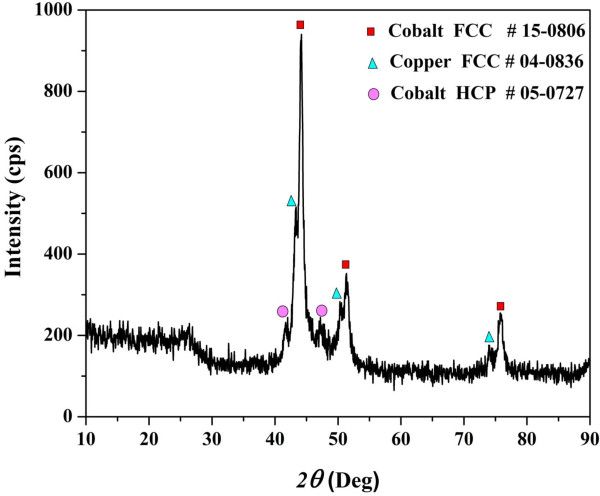
**XRD results for the obtained nanofibers.** The results indicate formation of FCC cobalt (JCPDS 15–0806, Sp.gr Fm3m (225)), HCP cobalt (JCPDS 05–0727, Sp.gr P63/mmc (194)), and copper (JCPDS 04–0836, Sp.gr Fm3m (225)).

Among the various analytical techniques, XRD received the most confidence to investigate the composition of the crystalline materials. The typical XRD pattern of the calcined powder is presented in Figure 2. Cobalt has more than one crystal structure, the most common ones are face-centered-cubic (FCC) and hexagonal close-packed (HCP) phases. The two phases of cobalt usually coexist at room temperature and are often difficult to be separated from each other. As shown in Figure 2, the strong diffraction peaks at 2*θ* values of 44.35°, 51.65°, 75.95°, 92.35°, and 97.75° corresponding to (111), (200), (220), (311), and (222) crystal planes indicate formation of FCC crystalline cobalt (JCPDS card no 15–0806). Moreover, HCP Co could be also detected as the standard peaks indicating that the formation of this phase can be seen at 41.72°, 44.73°, 47.63°, and 75.95° corresponding to crystal planes of (100), (002), (101), and (110), respectively (JCPDS card no 05–0727). It is noteworthy mentioning that the synthesis of Co nanostructures which is composed of two phases is already reported [40]. The formation of pure cobalt during the calcination of cobalt acetate in an inert atmosphere was explained in details in other studies [37,41,42]. Actually, abnormal decomposition of the acetate anion leads to form reducing gases (CO and H_2_), these gases are responsible about formation of the pure cobalt. Typically, formation of pure cobalt is conducted according to the following equations:

(2) $$\begin{array}{l} \mathrm{Co}{\left({\mathrm{CH}}_{3}\mathrm{COO}\right)}_{2}\;\cdot 4H_{2}O\to \mathrm{Co}\left(\mathrm{OH}\right)\left({\mathrm{CH}}_{3}\mathrm{COO}\right)\\ \;+3H_{2}O+{\mathrm{CH}}_{3}\mathrm{COOH} \end{array}$$

(3) $$\begin{array}{l} \mathrm{Co}\left(\mathrm{OH}\right)\left({\mathrm{CH}}_{3}\mathrm{COO}\right)\to \;0.5\mathrm{CoO}\\ \;+0.5{\mathrm{CoCO}}_{3}+0.5H_{2}O+0.5{\mathrm{CH}}_{3}{\mathrm{COCH}}_{3} \end{array}$$

(4) $${\mathrm{CoCO}}_{3}\;\to \mathrm{CoO}+{\mathrm{CO}}_{2}$$

(5) $$\mathrm{CoO}+\mathrm{CO}\to \mathrm{Co}+{\mathrm{CO}}_{2}$$

Concerning copper acetate, the XRD analysis indicates that this precursor has been decomposed to Cu metal, as the standard peaks of FCC copper can be observed at 2*θ* values of 43.3°, 50.5°, and 74.1° corresponding to (111), (200), and (220) crystal planes, respectively (JCPDS card no 04–0836). The formation of pristine copper from decomposition of copper acetate hydrate in an inert atmosphere was also proofed; the suggested stoichiometric equations were explained as follow [43]:

(6) $$\mathrm{Cu}{\left({\mathrm{CH}}_{3}\mathrm{COO}\right)}_{2}\cdot H_{2}O\to \mathrm{Cu}{\left({\mathrm{CH}}_{3}\mathrm{COO}\right)}_{2}+H_{2}O$$

(7) $$2\mathrm{Cu}{\left({\mathrm{CH}}_{3}\mathrm{COO}\right)}_{2}\to 2\mathrm{Cu}+3{\mathrm{CH}}_{3}{\mathrm{COOH}}_{2}+{\mathrm{CO}}_{2}+H_{2}+C$$

Additionally, a broad peak at 2*θ* of 26.3° corresponding to an experimental *d* spacing of 3.37 Å which indicates the presence of graphite-like carbon (*d* (002), JCPDS card no 41–1487) can be observed in the figure.

**Figure 3:**
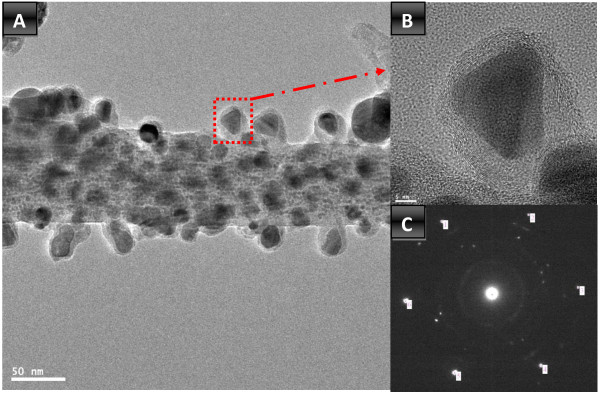
TEM, HRTEM and SAED images of HCP Co NPs.

**Figure 4:**
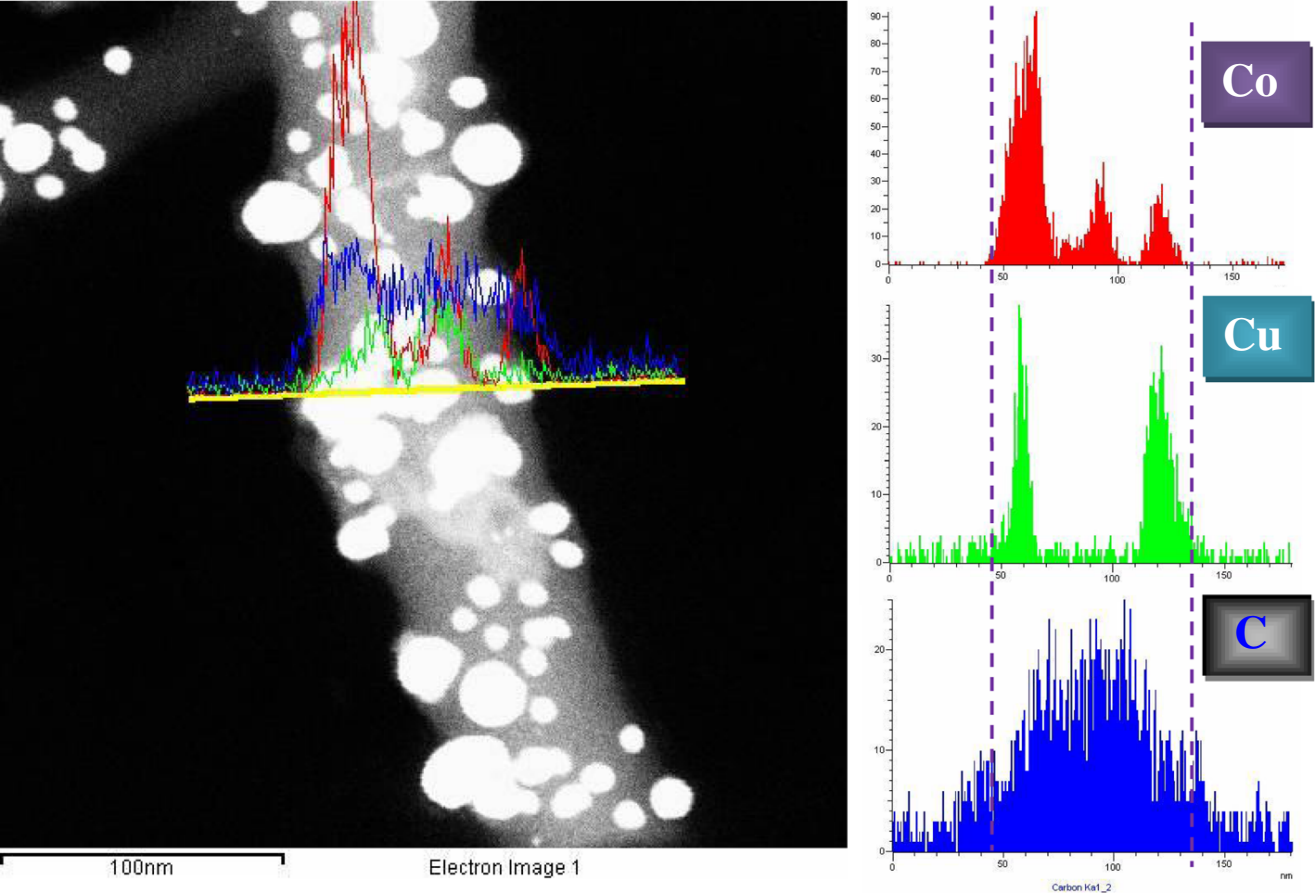
Line TEM EDX analysis of the produced nanofibers and the Co, Cu, and C distribution.

Observation about the inner structure of the synthesized nanofibers was done using TEM, high resolution transmission electron microscope (HRTEM), and selected area electron diffraction pattern (SAED) analysis; Figure 3 reveals the obtained results. Figure 3A shows that the TEM image of the synthesized nanofibers is decorated by crystalline metallic nanoparticles which support the FESEM image (Figure 1C). The HRTEM image in Figure 3B displaying one attached nanoparticles indicates that the attached nanoparticles have good crystallinity; moreover, these nanoparticles are sheathed by thin layer from good crystalline carbon. Interestingly, the SAED pattern of the chosen nanoparticles indicated hexagonal structure which confirms the formation of HCP cobalt and simultaneously supports the XRD results. Metallurgically, cobalt and copper can form solid solution alloys with a wide composition range. Therefore, TEM EDX analysis (Figure 4) was performed to understand the composition of the formed nanoparticles. As shown in the main image, the metallic nanoparticles have different sizes and also possess good crystallinity. Line EDX analysis was performed at a randomly selected line. As shown in concentration profiles, Co, Cu, and C were detected along with the line. Moreover, along the selected line, one can claim that there are pristine Co and Co/Cu alloy nanoparticles. It is noteworthy mentioning that the observed bare metallic nanoparticles in Figure 4 are sheathed in a carbon shell; however, due to the black and white image mode as well as the low carbon crystallinity, the carbon shell could not be observed. Aside from the normal TEM image (Figure 3) which explains that the metallic nanoparticles are covered by a thin layer of carbon, the performed line TEM EDX supports also this finding. As shown in Figure 4, carbon was detected beyond the distribution range of Co and Cu which was marked by two vertical dashed lines. In other words, this analysis indicates that carbon has a wide distribution compared to the metallic counterpart, and this confirms that the synthesized nanofibers are preserved against the chemicals by a shield of graphite layer.

**Figure 5:**
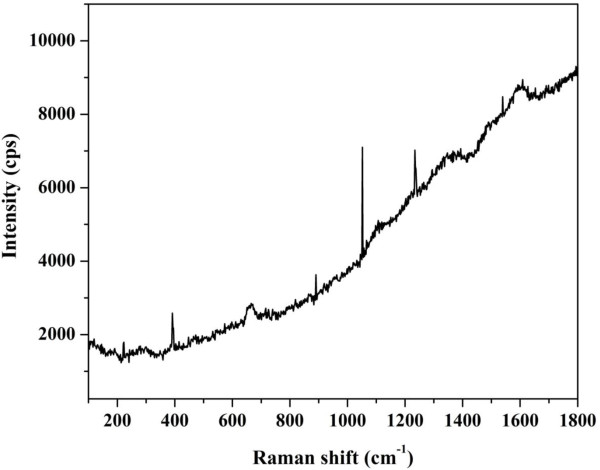
Raman shift for the obtained nanofibers.

Figure 5 shows the typical Raman spectra of the synthesized nanofibers. As shown in this figure, there is a clear peak at 1,335 cm^-1^, and this peak is called D peak which is present in all graphite-like carbons and originates from structural defects. The G peak corresponding to planar vibrations of carbon atoms in the graphite-like materials can be observed at 1,590 cm^-1^. The G peak is associated with the *E*_2g_ mode (stretching vibrations) in the basal-plane of graphite [44]. Another peak was detected at 195 cm^-1^, and this peak might be assigned as radial breathing mode (RBM). RBM corresponds to radial expansion-contraction of the carbon; its frequency *ν*_RBM_ depends on the diameter. Typical RBM range is 100 to 350 cm^-1^[45]. The other apparent peaks might be due to metallic counterpart, since these modes were not reported with graphite-like materials.

**Figure 6:**
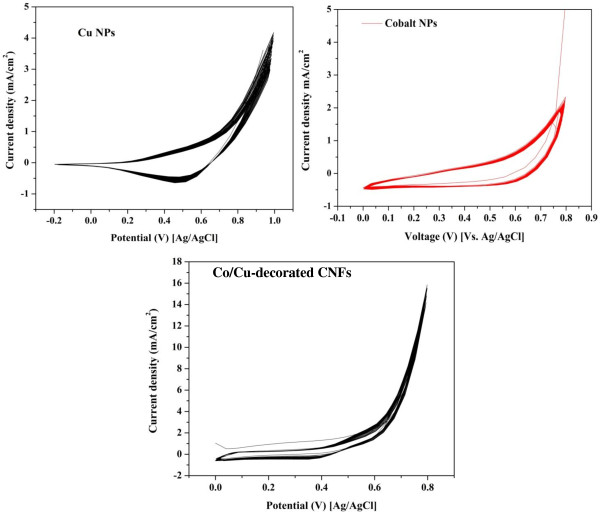
**Cyclic voltammograms of electrocatalyst, pristine cobalt, and copper nanoparticles.** Procedure was carried out in 1 M KOH for 50 cycles at a scan rate of 100 mV/s.

In contrast to the precious metals (especially Pt), usually the zero valent surface of the transition metals is not responsible on the electrocatalytic activity. For instance, in case of the nickel, NiOOH compound has to be synthesized on the surface to initiate the electrochemical activity [46]. The cyclic voltammetric behavior of cobalt and copper nanoparticles and the introduced nanofiber electrodes in 1.0 M KOH solution are shown in Figure 6. The utilized nanoparticles in this study were commercial products: cobalt nanoparticles <50 nm (Sigma Aldrich) and copper nanoparticles < 25 nm (nanoComposix, San Diego, CA, USA). Polarization was started by a potential scanning at a scan rate of 100 mV/s from 800 to 0 mV (vs. Ag/AgCl reference electrode) in the cathodic direction, and then the scan was reversed in the anodic direction back to 800 mV. Analogous to nickel [46], the appeared peaks in the voltammogram of the pristine copper nanoparticles can be considered as an activation of the anode surface to form the active CuOOH layer as follow [46]:

(8) $$\mathrm{Cu}+2{\mathrm{OH}}^{-}↔\;\mathrm{Cu}{\left(\mathrm{OH}\right)}_{2}+2e$$

(9) $$\mathrm{Cu}{\left(\mathrm{OH}\right)}_{2}+{\mathrm{OH}}^{-}↔\mathrm{CuOOH}+H_{2}O+e$$

Increasing the number of potential sweeps results in a progressive increase of the current density values of the cathodic peak because of the entry of OH^–^ into the Cu(OH)_2_ surface layer, which leads to the progressive formation of a thicker CuOOH layer corresponding to the Cu(OH)_2_/CuOOH transition [46]. On the other hand, it can be concluded from Figure 6 that Co has no surface activation which explains the known low electrocatalytic activity of this metal. It is noteworthy mentioning that high cobalt percentage was utilized in preparation of the introduced nanofibers to exploit the activity of Co in PVA graphitization to produce CNFs. Recently, supporting of the electrocatalysts on carbonaceous materials is carrying on to take the advantage of the adsorption capacity of the carbon and increase the active catalyst surface area [10,47]. The bottom panel in Figure 6 displays the obtained results in case of utilizing the introduced nanofibers. As shown in the figure, the presence of carbon and the nanofibrous morphology strongly enhance the current density compared to the pristine cobalt and copper nanoparticles.

**Figure 7:**
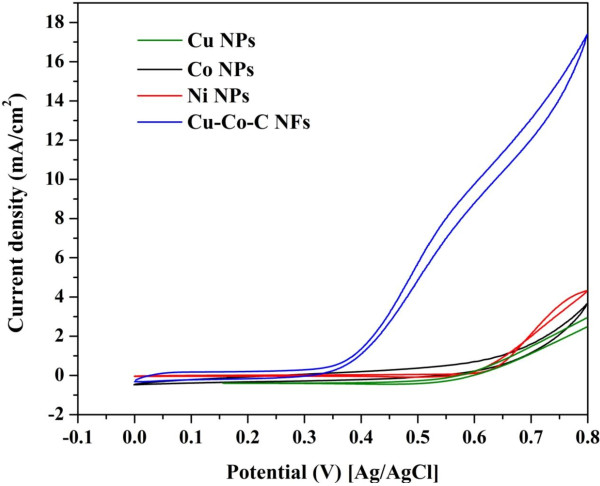
**Cyclic voltammograms of Co-Cu-C nanofibers, and Co, Ni, and Cu nanoparticles.** Amount of 2 M of methanol (scan rate 50 mV/s) was used.

Figure 7 displays the cyclic voltammetry for the introduced nanofibers and Co and Cu nanoparticles in presence of 2 M methanol solution. For comparison, nickel nanoparticles (<100 nm, Sigma Aldrich) have been also investigated due to the known electrocatalytic activity of nickel toward methanol electrooxidation. As shown in the figure, the introduced nanofibers have a significant performance compared to the metallic nanoparticles. The obtained good performance can be attributed to one or more from the following reasons: (a) influence of the nanofibrous morphology which, compared to nanoparticles, reveals better catalytic activity with the electron transfer-based reactions [25], (b) presence of carbon, (c) enhancement of the copper electrocatalytic activity due to alloying with Co, and (d) result of pre-adsorption of methanol molecules at Co sites by interaction of non-bonded electron pairs of the O atom of methanol with the partially vacant *d* orbital of the Co [48]. Moreover, the detected HCP cobalt, which according to our best knowledge was not investigated before as an electrocatalyst, may have an important contribution in the obtained good electrocatalytic activity.

**Figure 8:**
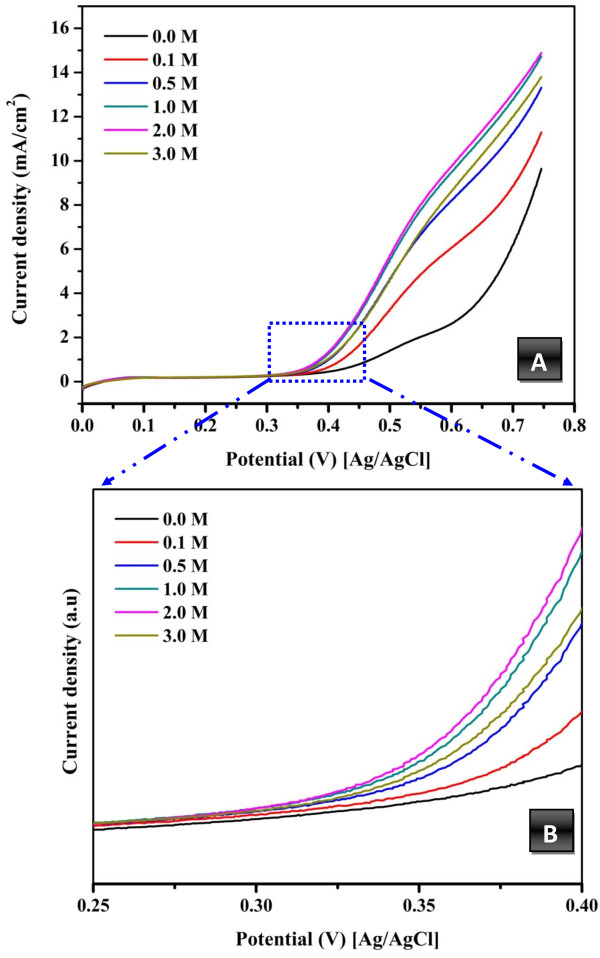
**Study the influence methanol concentration on the current density (A) and onset potential (B).** Scan rate of 50 mV/s at 25°C.

Figure 8A shows the influence of methanol addition on the obtained current density. As shown in the figure, the increase of methanol leads to the increase of the current densities, which indicates methanol oxidation on the surface of the introduced nanofibers. Another finding that can be observed is the optimum methanol concentration of 2 M which is consistent with many reported electrocatalysts. The onset potential is an important indicator among the invoked parameters to demonstrate the electrocatalytic activity. The onset potential indicates the electrode overpotential. In other words, the onset potential can be utilized to evaluate the efficacy of the electrocatalyst. In alcohol electrooxidation, more negative onset potential indicates high activity and less overpotential. As shown in the obtained results for the introduced electrocatalyst (Figure 8B), the corresponding onset potential of the introduced nanofibers is ~310 mV (vs. Ag/AgCl, i.e. ~510 mV vs. normal hydrogen electrode, NHE), and the obtained value is comparatively low compared to many reported materials [49]. This finding further supports the electrocatalytic performance of the introduced nanofibers. The decrease of the corresponding onset potential of the introduced nanofibers might be due to the influence of Co NPs which have been found as excellent CO-tolerant materials [11], and/or non-formation of CO as the corresponding oxidation peak is not observed at ~550 mV (vs. NHE i.e., 350 mV vs. Ag/AgCl).

The introduced Co/Cu electrocatalyst is novel, so the electrooxidation of the methanol can be explained based on previous literature [49,50] as follow:

1. Adsorption of the methanol on the introduced catalyst (M) and partial release of the protons

(10) $${\mathrm{CH}}_{3}\mathrm{OH}+M\to {\mathrm{MCH}}_{2}\mathrm{OH}+H^{+}+e$$

2. Further release of the protons

(11) $${\mathrm{MCH}}_{2}\mathrm{OH}+M\to \;M_{2}\mathrm{CHOH}+H^{+}+e$$

(12) $$M_{2}\mathrm{CHOH}+M\to \;M_{3}\mathrm{COH}+H^{+}+e$$

3. In the normal routes, the M_3_COH compound decomposes to produce MCO and proton as follows [50,51]:

(13) $$M_{3}\mathrm{COH}\to \mathrm{MCO}+2M+H^{+}+e$$

4. Later on, CO is oxidized by the OH group [50]; however, as aforementioned, CO oxidation on the surface of the introduced catalyst is not detected, so it is believed that reaction 13 does not occur. Instead, this reaction is taking place

(14) $$M_{2}\mathrm{COH}+H_{2}O\to \;3M+{\mathrm{CO}}_{2}+3H^{+}+3e$$

**Figure 9:**
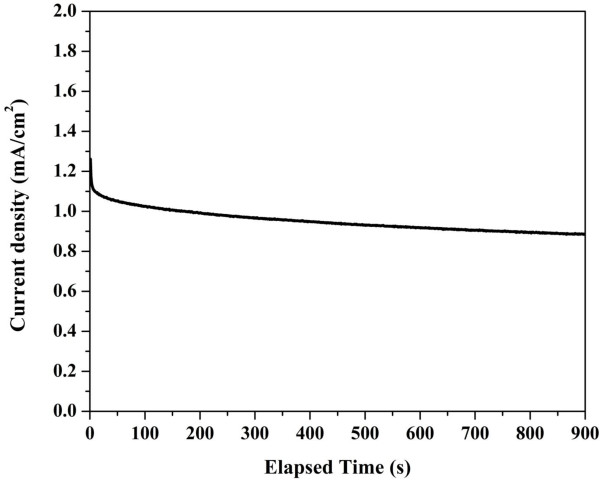
Constant voltage tests at a cell potential of 0.4 V and 2.0 M methanol.

Constant voltage tests were done at applied potential of 0.4 V in Figure 9. As shown in the figure, there is a sharp initial current drop, followed by a very slow decay. It is noteworthy mentioning that only one working electrode has been used in all the electrochemical analysis. The multiple use of the electrode did not affect the performance, which supports the good stability of the introduced catalyst. The obtained good stability might be explained by the thin graphite layer enveloping the introduced nanofibers.

**Figure 10:**
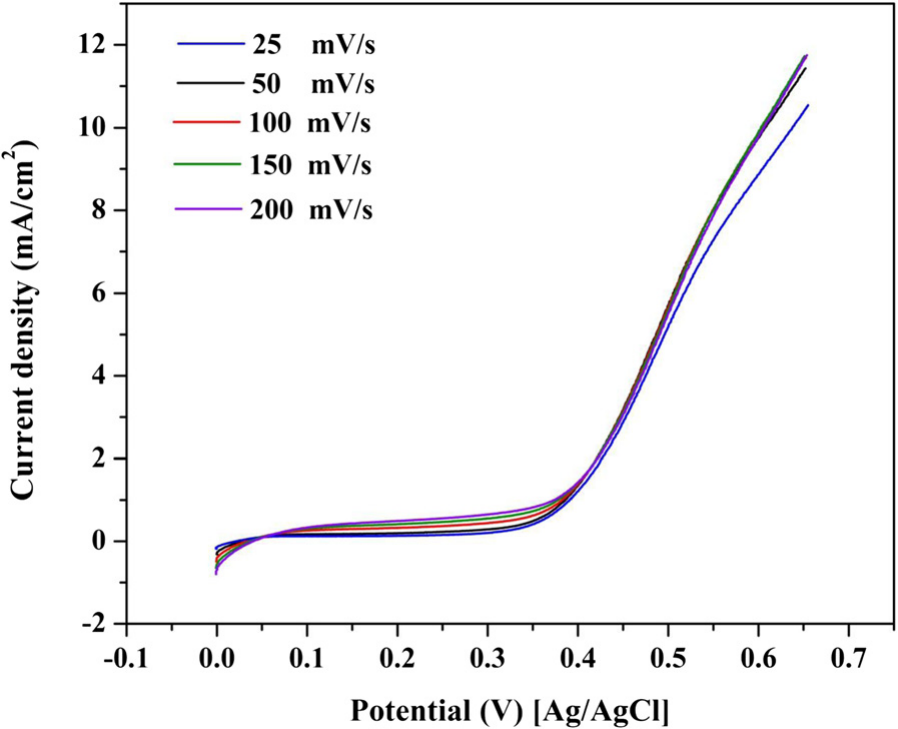
Linear sweep curves of methanol oxidation at different scan rates in 2.0 M methanol.

The effect of scan rate on the behavior of methanol oxidation at the introduced electrocatalyst was investigated (Figure 10). The study of the scan rate is helpful to investigate the rate-controlling step. For instance, the linear variation of the current densities with respect to the scan rate indicates that the process of electrooxidation of methanol has the characteristics of a diffusion-controlled process [52]. As shown in Figure 10, the scan rate has almost no influence on the current density, which indicates that the rate-controlling step is another process rather than the diffusion which might be investigated in a future study.

## Conclusions

The electrospinning process can be efficiently utilized to produce carbon nanofibers decorated by metallic nanoparticles. Cobalt/copper-decorated carbon nanofibers can be produced by electrospinning of a sol–gel composed of cobalt acetate tetrahydrate, copper acetate monohydrate and poly(vinyl alcohol). Calcination of the electrospun mats in argon atmosphere produces Co/Cu-decorated CNFs. The produced decorated nanofibers can be utilized as electrocatalyst for methanol oxidation; the corresponding onset potential and current density are satisfactory. Moreover, good stability is expected because the metallic nanoparticles are sheathed inside the thin graphite layer. Overall, this study introduces new methodology to produce metal-decorated CNFs based on PVA. The proposed decorated nanofibers can be further modified to be more efficient non-precious electrocatalysts for fuel cell applications.

## Competing interests

The authors declare that they have no competing interests.

## Authors’ contributions

NAB fabricated the introduced nanofibers, carried out the characterization techniques, and the electrochemical measurements, and wrote the paper. MHE helped in writing the paper and performing the characterization process. SSE helped in the paper writing and electrochemical measurements. HYK helped in the nanofibers synthesis and electrochemical measurements. All authors read and approved the final manuscript.
